# Maternal seafood intake, dietary contaminant exposure, and risk of juvenile idiopathic arthritis: exploring gene-environment interactions

**DOI:** 10.3389/fimmu.2024.1523990

**Published:** 2025-01-14

**Authors:** Vilde Øverlien Dåstøl, Kristine Løkås Haftorn, Hamid Khoshfekr Rudsari, Piotr Pawel Jaholkowski, Ketil Størdal, Siri Eldevik Håberg, Clarice R. Weinberg, Lisa G. Rider, Ole A. Andreassen, Anne Lise Brantsæter, Ida Henriette Caspersen, Helga Sanner

**Affiliations:** ^1^ Department of Rheumatology, Oslo University Hospital, Oslo, Norway; ^2^ Institute of Clinical Medicine, Faculty of Medicine, University of Oslo, Oslo, Norway; ^3^ Center for Precision Psychiatry, Division of Mental Health and Addiction, Oslo University Hospital, and Institute of Clinical Medicine, University of Oslo, Oslo, Norway; ^4^ KG Jebsen Centre for Neurodevelopmental Disorders, University of Oslo and Oslo University Hospital, Oslo, Norway; ^5^ Department of Pediatric Research, Institute of Clinical Medicine, Faculty of Medicine, University of Oslo, Oslo, Norway; ^6^ Department of Pediatric and Adolescent Medicine, Oslo University Hospital, Oslo, Norway; ^7^ Centre for Fertility and Health, Norwegian Institute of Public Health, Oslo, Norway; ^8^ Department of Global Public Health and Primary Care, University of Bergen, Bergen, Norway; ^9^ Biostatistics and Computational Biology Branch, National Institute of Environmental Health Sciences, Research Triangle Park, NC, United States; ^10^ Environmental Autoimmunity Group, Clinical Research Branch, National Institute of Environmental Health Sciences, National Institutes of Health, Bethesda, MD, United States; ^11^ Center for Precision Psychiatry, Division of Mental Health and Addiction, Oslo University Hospital, Oslo, Norway; ^12^ Department of Food Safety and Centre for Sustainable Diets, Norwegian Institute of Public Health, Oslo, Norway; ^13^ Oslo New University College, Oslo, Norway

**Keywords:** juvenile idiopathic arthritis (JIA), MoBa, fish, contaminants, heavy metals, polygenic risk score, gene-environment interaction, sex differences

## Abstract

**Objectives:**

Juvenile idiopathic arthritis (JIA) originates from a complex interplay between genetic and environmental factors. We investigated the association between seafood intake and dietary contaminant exposure during pregnancy and JIA risk, to identify sex differences and gene-environment interactions.

**Methods:**

We used the Norwegian Mother, Father, and Child Cohort Study (MoBa), a population-based prospective pregnancy cohort (1999–2008). JIA patients were identified through the Norwegian Patient Registry, with remaining mother-child pairs serving as controls. We assessed maternal seafood intake and dietary contaminants typically found in seafood using a food frequency questionnaire completed during pregnancy, mainly comparing high (≥90^th^ percentile, P90) vs low (<P90) intake. Multivariable logistic regression calculated adjusted odds ratios (aOR), including sex-stratification analyses. A polygenic risk score (PRS) for JIA was used in a subsample to assess gene-environment interactions.

**Results:**

We identified 217 JIA patients and 71,884 controls. High vs low maternal intake of lean/semi-oily fish was associated with JIA (aOR 1.51, 95% CI 1.02-2.22), especially among boys (aOR 2.13, 95% CI 1.21-3.75). A significant gene-environment interaction was observed between total fish intake and PRS, with high fish intake associated with JIA primarily in those with low PRS (p<0.03). We found no associations between high vs low exposure to other types of seafood or environmental contaminants and JIA.

**Conclusions:**

We found a modestly increased risk of JIA associated with high intake of lean/semi-oily fish during pregnancy, not explained by estimated exposure to dietary contaminants. Our data suggest a more pronounced association in children with a lower genetic predisposition for JIA.

## 1 Introduction

Juvenile idiopathic arthritis (JIA), the most common inflammatory rheumatic disease of childhood, manifests as arthritis before the age of 16 years which persists more than six weeks, and without an apparent cause. It consists of seven heterogeneous subgroups, reflecting the complex interplay between genetic predisposition and environmental influences that contribute to the diverse clinical manifestations (1). Known genetic variants are estimated to account for 13-25% of the risk for JIA, while the remaining risk is attributed to environmental factors and their interaction with genetic predisposition (1, 2). Limited high-quality data and modest sample sizes have constrained prior attempts to pinpoint environmental risk and protective factors (3). Furthermore, despite JIA being more prevalent in girls than in boys (4), few studies have investigated this sex disparity, which is important for understanding the underlying pathomechanisms of disease development.

Diet is an example of an environmental factor that remains underexplored in relation to JIA risk (3). Results from a Swedish prospective cohort study showed that fish intake more than once per week during pregnancy and the first year of life was associated with increased risk of JIA, which was mainly attributed to high heavy metal exposure (5).

Among the environmental contributors, heavy metals like mercury and cadmium, and persistent organic pollutants (POPs), have emerged as potential triggers of autoimmunity (6–9). Mercury is associated with subclinical autoimmunity in humans through the production of autoantibodies and cytokines (10–13), while in individuals with a genetic predisposition, cadmium may exacerbate autoimmunity (14) and increase the risk of rheumatoid arthritis (RA) (15, 16). Furthermore, exposure to POPs has also been linked to autoimmune diseases, with research suggesting increased risk of celiac disease, especially in girls (8), and of RA (17).

Diet serves as a major source of these contaminants (18), with seafood being a significant contributor to mercury (19) and shellfish contributing to cadmium exposure (20). Individuals consuming high amounts of seafood are also at greater risk of POPs exposure (21, 22). It has been suggested that diseases with a sex disparity should be investigated for environmental risk factors like contaminant exposure, as differences in vulnerability and susceptibility between the sexes may account for the prevalence disparities (23).

Our primary aim was to explore the association between seafood intake and dietary environmental contaminant exposure during pregnancy and JIA risk. Secondary aims included exploring sex disparities and possible interactions between seafood intake and genetic predisposition to JIA.

## 2 Material and methods

### 2.1 Study population and design

We used data from the Norwegian Mother, Father, and Child Cohort Study (MoBa), which was linked by national identification (ID) numbers to the individual records in the following population-based health registers: the Norwegian Patient Registry (NPR) and the Medical Birth Registry of Norway (MBRN).

MoBa is a population-based pregnancy cohort study conducted by the Norwegian Institute of Public Health. Participants were recruited from all over Norway from 1999-2008. Of those invited to participate, 41% of women consented. The cohort includes approximately 114,500 children, 95,200 mothers, and 75,200 fathers. The current study is based on version 12 of the quality-assured data files released for research in 2019 (24). Genotype data was available for a subsample of 51,804 children, which is further described under “Genotyping Data, Polygenic Risk Score (PRS) for JIA”.

**Figure 1:**
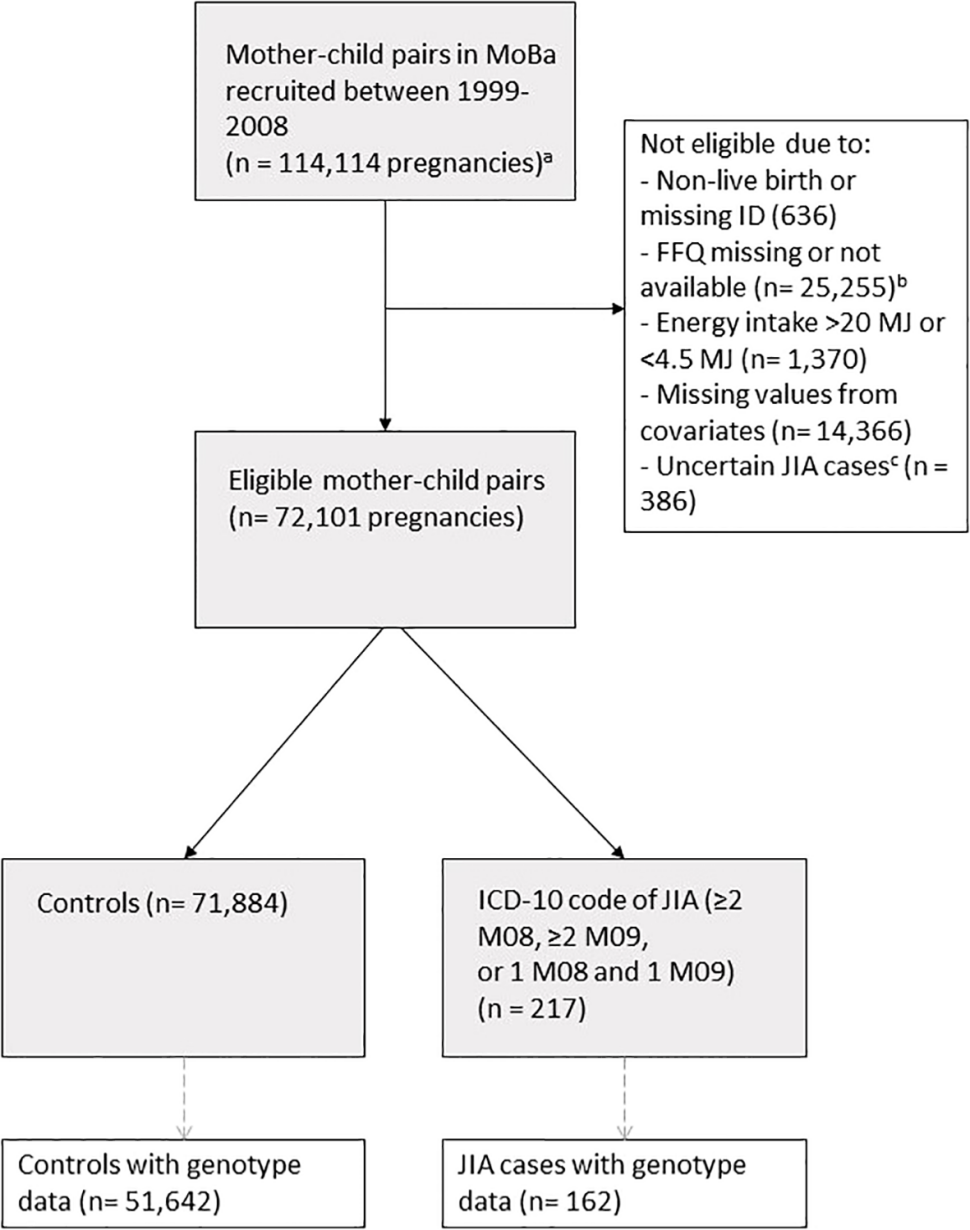
Flowchart of study population with exclusion criteria. ^a^ This number includes siblings. ^b^ The MoBa FFQ was introduced in 2002. ^c^ We excluded all controls with a single ICD-10 code (M08 or M09) to rule out potential JIA cases, except for those who received their first code in 2021. For these cases, we accepted a single relevant ICD-10 code, acknowledging that they might have had only one doctor visit before our latest NPR update in 2022.

Three questionnaires were sent to the mothers during pregnancy, the second being a semi-quantitative food frequency questionnaire (FFQ). The FFQ was distributed in gestational week 22 and covered the average intake of 255 food items and beverages during the first half of pregnancy (25). The MoBa FFQ has been validated and found to be a reliable tool to estimate intake of nutrients and foods during pregnancy, including various types of fish and seafood (26, 27). The FFQ was introduced in March 2002 and all pregnancies recruited between 2002 and 2008 are included in our study. **Figure 1** outlines the flow of subject for inclusion in our study from the MoBa cohort.

### 2.2 Outcome

The Norwegian Patient Registry (NPR) contains data with personal ID numbers from all Norwegian public hospitals and specialists with public funding from 2008. We defined a JIA case as having at least two International Classification of Diseases (ICD)-10 codes (≥2 M08, ≥2 M09, or 1 M08 *and* 1 M09). We recently validated this case definition and have found a positive predictive value of 93.4% (28). For cases where the child received their first ICD-10 code in 2021, we accepted a single relevant ICD-10 code (M08 or M09), recognizing that there might have been only one doctor visit before we received our latest updates from NPR in December 2021. Controls were defined as live births that were non-JIA cases. We excluded children with only one registration of ICD-10 code M08 or M09 between 2008-2020 because they might have JIA.

### 2.3 Exposure variables: intake of seafood and environmental contaminants

We estimated maternal seafood intake and the exposure to dietary contaminants by the FFQ that was developed and validated for pregnant women in MoBa (25).

Food frequencies reported in the FFQ were converted to food amounts (grams/day) using FoodCalc and the Norwegian food table (26). Seafood intake was allocated into five variables, of which three were strictly related to fish intake: 1) oily fish (more than 8% fat, such as herring, mackerel, salmon), 2) lean/semi-oily fish (up to 8% fat, such as cod, haddock, saithe), and 3) total fish (total amount of oily fish and lean/semi-oily fish). In addition, we included 4) shellfish intake (capturing crab, shrimp, and mussels), and 5) total seafood (total fish and shellfish, including fish liver, roe, and fish liver/roe spread). We converted these continuous variables (grams/day) into categorical variables in the following way:

We categorized the seafood variables into high intake, defined as equal to or exceeding the 90th percentile (≥P90) of the population, and low intake, defined as less than 90th percentile (<P90). In secondary analyses, the five seafood variables were also divided into quintiles with the lowest group serving as reference. Lastly, because the Norwegian Directorate of Health recommends between 300-450 grams of fish each week (29), an exposure variable was also set at ≥300 grams of fish per week, which was compared to intake <300 grams/week.

The exposure to dietary environmental contaminants was estimated by combining consumption data from the FFQ with concentrations of contaminants in Norwegian food, based on data across various Nordic studies and databases, with the mean or median values from these studies used for the estimation of dietary contaminant exposure. The food contamination data spans several years, corresponding with the period when the FFQ was completed, and is described elsewhere (30, 31). Dietary contaminant exposure was categorized into two main groups: 1) heavy metals and 2) persistent organic pollutants (POPs). Heavy metals included a) mercury, and b) cadmium, while POPs included c) dioxins and dioxin-like (dl) compounds, and d) non-dioxin-like (non-dl) polychlorinated biphenyls (using PCB-153 as a proxy). The exposure to dioxins and dl-compounds is expressed as toxic equivalents (TEQ) when assessing their combined effect (32). The dietary contaminant variables were calculated per kilogram of pre-pregnancy body weight (kg bw), which was self-reported. We analyzed high vs. low intake and across quintiles as defined above.

### 2.4 Covariates and confounders

Potential confounding factors included maternal education, and parity (categorical variables); maternal age, pre-pregnancy BMI, daily energy intake (continuous variables); and maternal history of inflammatory rheumatic disease (see definition below), parental smoking status, and maternal supplement use during pregnancy (e.g., fish oil, vitamin D, folate) as dichotomous variables (yes/no). Associations with lean/semi-oily and oily fish were mutually adjusted due to their correlated intake.

When analyzing dietary environmental contaminants, we included the child’s birth year from the Medical Birth Registry as a possible confounder because contaminant levels in fish may have varied over the years, and because the cumulative risk of JIA increases with the child’s age. Information about region of birth was also obtained from the Medical Birth Registry.

Mother’s history of inflammatory rheumatic diseases was obtained via linkage to NPR and included following ICD-10 codes: M05, M06, M07, M08, M09, M30, M31, M32, M33, M34, M35, M45, M46, and L94.

### 2.5 Genotyping data, polygenic risk score for JIA

In MoBa, umbilical cord blood samples were collected at birth and DNA was stored at the Norwegian Institute of Public Health (33). Genotyping was carried out over several years through various research projects (34). MoBaPsychGen genotype quality control (QC) pipeline was developed to manage the complex relationships within the cohort. This pipeline includes steps for pre-imputation QC, phasing, imputation, and post-imputation QC, and it accounts for array and batch effects (35).

We focused on individuals of European ancestry, identified by visually comparing the first seven genetic principal components (PCs) to those from unrelated samples in the 1000 Genomes phase 1 project (35). Related individuals with a kinship coefficient >0.05 had one member excluded, prioritizing the retention of JIA cases, with other exclusions made randomly.

To estimate the genetic risk for JIA, we calculated PRSs using data from a genome-wide association study (GWAS) of JIA (36). The calculation was done using PRSice version 2.3.3 (37), applying different P-value thresholds as 5E-8, 1E-6, 1E-5, 1E-4, 1E-3, 1E-2, 5E-2, 1E-1, 5E-1, and 1. We then extracted the first PC of PRSs across all P-value thresholds, following a widely used method (38). The standardized PRS was then converted into a binary variable with cut-off at 0, of which the PRS <0 was regarded as “low”, whereas the PRS ≥0 was regarded as “high”.

### 2.6 Statistical analysis

Stata V.17.0 statistical software (StataCorp) and R version 4.2.3 (39) were used to conduct all statistical analyses. Characteristics of high vs low consumers of fish were reported as mean (SD) or median (IQR), as appropriate for continuous variables and by distribution differences (counts and percentages) for categorical variables. We used multiple logistic regression to examine the associations between seafood intake, dietary environmental contaminant exposure and risk of JIA. All associations are reported as odds ratios (OR) with 95% confidence intervals (CI), and as adjusted ORs (aOR) when adjusted for possible confounding factors listed above. The number of subjects with missing values on covariates was low for both cases (n=40, 18%) and controls (n=14,366, 20%), and all estimates are therefore based on complete case analyses. All analyses were further stratified by sex. In a sensitivity analysis, we included the region of birth (South-East, West, Middle and North), and thus presumably the region where the pregnancy took place, as a possible confounder because research shows a two-fold increased incidence of JIA in northern compared to southern Norway (28) and reports of geographical variations in fish intake (40).

To assess potential interactions between fish intake and genetic predisposition to JIA, we conducted multiple logistic regression analyses with an interaction term between fish intake and PRS. We included the same variables as in the main model except maternal history of inflammatory rheumatic disease to avoid over-adjustment. The Wald test was used to assess statistical significance of an interaction, and a p-value <0.05 was regarded as significant. We further investigated the interaction between fish intake and PRS by calculating the products of fish intake and dummy variables of each PRS group and replacing the interaction term in the multiple logistic regression with those products. This allowed us to estimate the association between fish intake and JIA in the low and high PRS groups separately. We used this model to visualize the relationship between fish intake and JIA in both groups predicting JIA risk in a simulated dataset of n = 200. As an additional test for interactions, we applied a case-only analysis by testing for associations between seafood intake and PRS in the cases only (41).

## 3 Results

### 3.1 Study sample characteristics

Our final analytical sample included 72,110 mother-child pairs; 217 children with JIA were identified (**Figure 1**). Of JIA cases, there were 139 (64.1%) girls and 78 (35.9%) boys. The median weekly maternal fish intake was 218 grams. Baseline characteristics categorized by high (≥P90) vs. low (<P90) total fish intake are shown in **Table 1**.

**Table 1:** Baseline characteristics categorized by high and low total fish intake in 72,101 MoBa participants 2002-2008.

Characteristics	High total fish intake (P≥90)*	Low total fish intake (<P90) *
Population	7,209 (10.0)	64,892 (90.0)
Maternal age at delivery, years, mean (SD)	31.0 (4.8)	30.3 (4.5)
Maternal education		
High school or less	2,722 (37.8)	21,839 (33.7)
College, up to 4 years	2,675 (37.1)	27,202 (42.0)
College, more than 4 years	1,812 (25.1)	15,851 (24.4)
Maternal pre-pregnancy BMI, mean (SD)	24.0 (4.4)	24.1 (4.3)
Maternal parity		
0	3,097 (43.0)	29,714 (46.0)
1	2,487 (34.5)	23,185 (36)
2 or more	1,624 (22.5)	11,993 (18.5)
Inflammatory rheumatic disease in mother		
Yes	209 (2.9)	1,853 (2.9)
No	7,000 (97.1)	63,039 (97.1)
Maternal daily caloric intake, kcal, median (IQR)	2462 (2053, 2939)	2207 (1866, 2620)
Maternal smoking status during pregnancy		
Yes	642 (8.9)	5,048 (7.8)
No	6,567 (91.1)	59,844 (92.2)
Paternal smoking status		
Yes	1448 (20.1)	12,624 (19.5)
No	5761 (79.9)	52,268 (80.6)
Dietary supplement use during pregnancy		
Yes	6,167 (85.6)	56,032 (86.4)
No	1,042 (14.5)	8,860 (13.7)
Region of birth		
South-East	3,365 (46.7)	35,794 (55.2)
West	1,955 (27.1)	16,132 (24.9)
Middle	1,207 (16.7)	9,296 (14.3)
North	682 (9.5)	3,670 (5.7)

*High is defined as equal to or above 90^th^ percentile, while low is defined as below 90^th^ percentile.

Numbers are n (%), mean (SD) or median (IQR).

### 3.2 Seafood intake and JIA

High vs low intake of lean/semi-oily fish during pregnancy was associated with JIA (aOR 1.51, 95% CI 1.02-2.22) (**Table 2**). After adjusting for region of birth, the confidence interval included 1 (aOR 1.45, 95% CI 0.99-2.18) (**Supplementary Table 1**). Additional results with region of birth as a covariate are presented in **Supplementary Table 1-Supplementary Table 2**. We found no other evidence of associations between high vs low intake of other seafood variables and JIA risk (**Table 2**).

**Table 2:** Overall and sex-stratified associations between high vs. low seafood intake and JIA.

	All (controls n= 71,884, JIA cases n= 217)		Boys (controls n= 36,784 and JIA cases n= 78)		Girls (controls n= 35,100, JIA cases n= 139)	
	Unadjusted OR (95% CI)	aOR^a^ (95% CI)	Unadjusted OR (95% CI)	aOR^a^ (95% CI)	Unadjusted OR (95% CI)	aOR^a^ (95% CI)
High total fish intake						
<90^th^ percentile	Ref	Ref	Ref	Ref	Ref	Ref
≥90^th^ percentile (≥423.5 grams/week)	1.02 (0.65-1.58)	1.02 (0.65-1.59)	1.78 (0.98-3.23)	1.80 (0.98-3.31)	0.63 (0.32-1.24)	0.63 (0.32-1.24)
High lean/semioily fish intake						
<90^th^ percentile	Ref	Ref	Ref	Ref	Ref	Ref
≥90^th^ percentile (≥249.5 grams/week)	**1.50** **(1.03-2.20)**	**1.51** **(1.02-2.22)**	**2.13** **(1.21-3.75)**	**2.07** **(1.17-3.66)**	1.18 (0.70-1.99)	1.21 (0.72-2.06)
High oily fish intake						
<90^th^ percentile	Ref	Ref	Ref	Ref	Ref	Ref
≥90^th^ percentile (≥156 grams/week)	0.81 (0.50-1.32)	0.80 (0.49-1.31)	1.45 (0.76-2.74)	1.36 (0.71-2.62)	0.49 (0.23-1.04)	0.49 (0.23-1.06)
High shellfish intake						
<90^th^ percentile	Ref	Ref	Ref	Ref	Ref	Ref
≥90^th^ percentile (≥65 grams/week)	1.12 (0.73-1.71)	1.14 (0.74-1.74)	**1.83** **(1.01-3.33)**	**1.86** **(1.02-3.38)**	0.76 (0.41-1.41)	0.78 (0.42-1.44)
High seafood intake						
<90^th^ percentile	Ref	Ref	Ref	Ref	Ref	Ref
≥90^th^ percentile (≥492 grams/week)	0.91 (0.58-1.49)	0.92 (0.57-1.46)	1.62 (0.87-3.00)	1.64 (0.88-3.07)	0.56 (0.27-1.13)	0.55 (0.27-1.14)

a Adjusted for maternal age, education, pre-pregnancy BMI, parity, daily caloric intake, history of inflammatory rheumatic disease in mother, parental smoking status during pregnancy and supplement use during pregnancy. When lean/semioily fish is the main exposure, it is also adjusted for oily fish intake, and vice-versa. Bold text indicates statistically significant results.

After sex-stratification, we found an association with lean/semi-oily fish intake among boys (aOR 2.07, 95% CI 1.17-3.66), but not in girls (**Table 2** and **Supplementary Table 1**). Similarly, high shellfish intake was associated with increased risk among boys (aOR 1.86, 95% CI 1.02-3.38), but not girls (**Table 2**). Additionally, consuming fish ≥300 vs. <300 grams/week during pregnancy, regardless of fat content, was linked to higher odds of JIA in boys (aOR 1.92, 95% CI: 1.22-3.04), but not in girls (**Supplementary Table 3**). When analyzing by quintiles, no other convincing evidence of associations were observed (**Supplementary Table 4**).

### 3.3 Interactions between fish intake and polygenic risk score

The following results are based on a smaller sample than our main analyses (controls n= 51,642, JIA case n= 162) due to lack of genetic data on all observations. To account for this, we ran the main analyses on the smaller dataset as a sensitivity analysis, with the results provided in **Supplementary Table 5.**

**Figure 2:**
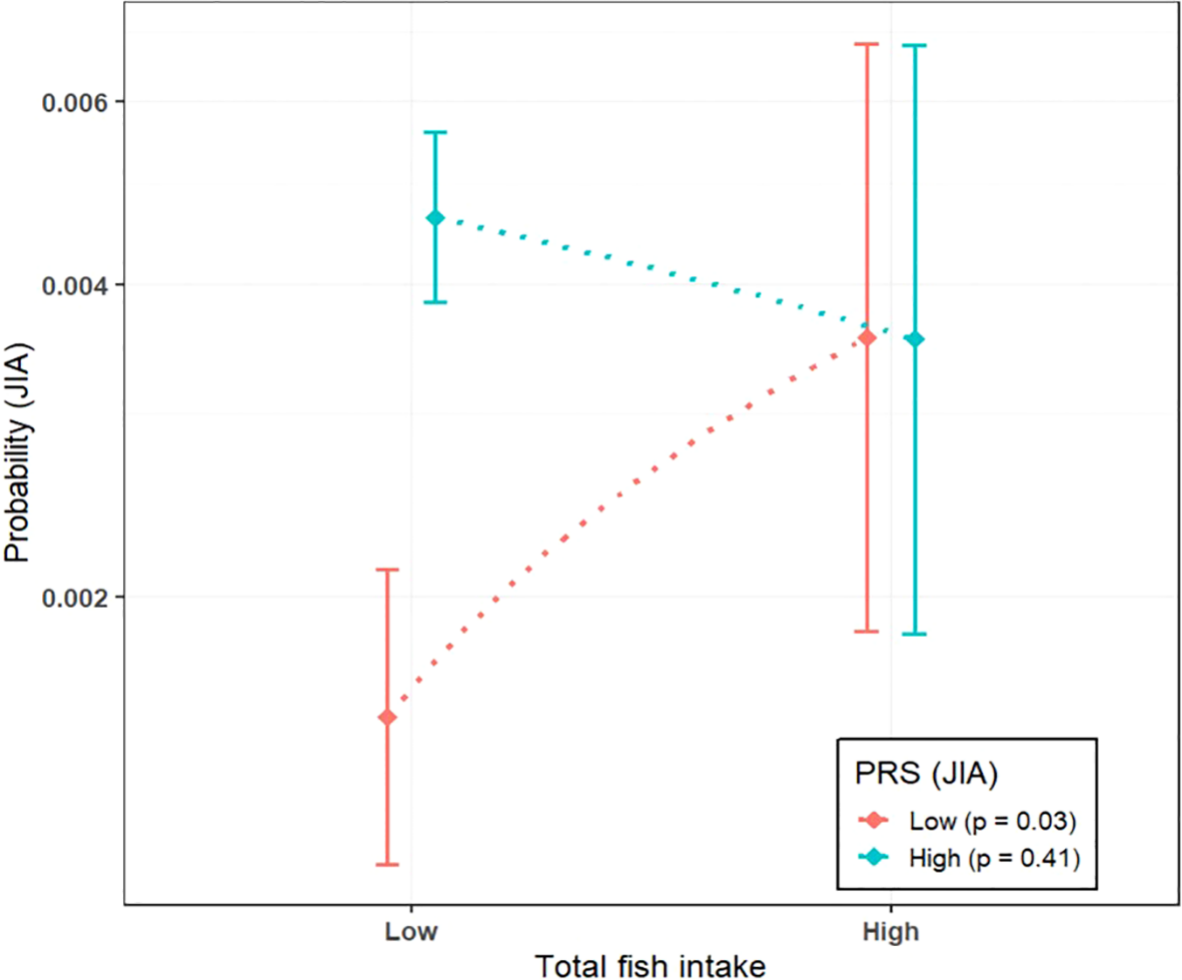
Association between total fish intake and JIA risk grouped by high (=>0) and low (<0) polygenic risk score (PRS) for JIA. P-values indicate the significance of the associations between fish intake and JIA risk within each PRS group.

We found evidence of an interaction between total fish intake and PRS (aOR 0.33, 95% CI 0.12-0.90, p-value 0.03), but not with the other seafood variables (**Supplementary Table 6**). The association between total fish intake and JIA was only apparent in the low PRS group (aOR 2.26, 95% CI 1.08-4.71) (**Table 3** and **Figure 2**). Furthermore, we also found an association between lean/semi-oily fish and JIA in the low PRS group (aOR 2.23, 95% CI 1.06-4.66), but not with the other seafood variables (**Table 3** and **Supplementary Figure 1-Supplementary Figure 2**). A case-only design was used to test the interaction between fish intake and PRS, which further confirmed the findings from the case-control analyses: the high total fish intake was negatively associated with PRS in the cases, whereas none of the other seafood variables reached statistical significance (**Supplementary Table 7**).

**Table 3:** Associations between high seafood intake and JIA risk in groups of high or low genetic risk (PRS of JIA).

Exposure	PRS group^a^	aOR^b^ (95% CI)
High total fish	Low	**2.26 (1.08-4.71)**
	High	0.75 (0.38-1.49)
High lean/semioily fish	Low	**2.23 (1.06-4.66)**
	High	1.14 (0.63-2.05)
High oily fish	Low	0.65 (0.20-2.11)
	High	0.84 (0.43-1.61)
High shellfish	Low	1.65 (0.74-3.71)
	High	1.38 (0.81-2.36)
High seafood	Low	1.67 (0.74-3.77)
	High	0.82 (0.43-1.58)

a The standardized PRS was converted into a binary variable with cut-off at 0, of which the PRS <0 was regarded as “low”, whereas the PRS ≥0 was regarded as “high”.

b Adjusted for: maternal age, education, pre-pregnancy BMI, parity, daily caloric intake, parental smoking status during pregnancy, supplement use during pregnancy, high PRS and PCs 1-10. When lean/semioily fish is the main exposure, it is also adjusted for oily fish intake, and vice-versa. (controls n= 51,642, JIA cases n= 162).Bold text indicates statistically significant results.

### 3.4 Estimated environmental contaminants and JIA

We found no evidence of associations between estimated dietary intake of environmental contaminants and risk of JIA, whether analyzed by high vs. low intake (**Table 4**) or by quintiles (**Supplementary Table 8**).

**Table 4:** Overall and sex-stratified associations between high vs. low dietary contaminant exposure^a^ and JIA.

	All (controls n= 71,884, JIA cases n= 217)		Boys (controls n= 36,784 and JIA cases n= 78)		Girls (controls n= 35,100, JIA cases n= 139)	
	Unadjusted OR (95% CI)	aOR^b^ (95% CI)	Unadjusted OR (95% CI)	aOR^b^ (95% CI)	Unadjusted OR (95% CI)	aOR^b^ (95% CI)
Mercury						
<90^th^ percentile	Ref	Ref	Ref	Ref	Ref	Ref
≥90^th^ percentile (≥0.3 ug/kg bw/week)	0.91 (0.58-1.45)	0.92 (0.57-1.46)	1.17 (0.58-2.35)	1.22 (0.60-2.48)	0.77 (0.42-1.44)	0.76 (0.41-1.42)
Cadmium						
<90^th^ percentile	Ref	Ref	Ref	Ref	Ref	Ref
≥90^th^ percentile (≥2.1 ug/kg bw/week)	1.39 (0.94-2.06)	1.42 (0.94-2.14)	1.47 (0.78-2.79)	1.51 (0.77-2.99)	1.35 (0.82-2.21)	1.36 (0.80-2.29)
Dioxins and dioxin-like (dl) compounds						
<90^th^ percentile	Ref	Ref	Ref	Ref	Ref	Ref
≥90^th^ percentile (≥7.5 pg TEQ/kg bw/week)	0.86 (0.54-1.38)	0.83 (0.51-1.34)	1.46 (0.77-2.77)	1.45 (0.74-2.82)	0.56 (0.27-1.14)	0.52 (0.25-1.08)
Non-dioxin-like PCBs (PCB-153)						
<90^th^ percentile	Ref	Ref	Ref	Ref	Ref	Ref
≥90^th^ percentile (≥13.2 pg/kg bw/week)	0.91 (0.58-1.45)	0.90 (0.56-1.43)	1.03 (0.49-2.14)	1.02 (0.48-2.14)	0.85 (0.47-1.54)	0.83 (0.46-1.51)

a Contaminants were estimated by combining consumption data from the FFQ with concentrations of contaminants in Norwegian food.

b Adjusted for maternal age, education, pre-pregnancy BMI, parity, daily caloric intake, history of inflammatory rheumatic disease in mother, parental smoking status during pregnancy, supplement use during pregnancy and the child’s birth year.

After sex-stratification, we found a positive association between non-dl PCBs and JIA in boys (aOR 2.24, 95% CI 1.03-4.86), when comparing a dietary exposure corresponding to the 4^th^ quintile to the 1^st^ quintile (**Supplementary Table 8**). Among girls, being in the 5^th^ quintile of either dl-compound or non-dl PCB intake, was negatively associated with risk of JIA (aOR 0.40, 95% CI 0.20-0.79 and aOR 0.44, 95% CI 0.23-0.83; **Supplementary Table 8**).

## 4 Discussion

In this large population-based study, we found a modestly increased risk of JIA associated with high maternal intake of lean/semi-oily fish (approximately 250 grams or more per week) during pregnancy. No clear associations were found between JIA and overall maternal intake of fish, oily fish, shellfish, or seafood intake. Sex-stratified analyses suggested a stronger positive association between high maternal seafood intake and JIA risk in boys. For instance, an intake of >300 grams of fish per week as recommended by the Norwegian Directorate of Health (29), was linked to increased risk of JIA in boys but not in girls. We observed no clear associations with estimated maternal dietary contaminant exposures. The risk associated with total fish intake depended on genetic predisposition: high fish intake significantly affected JIA risk only in individuals with a low genetic predisposition to JIA.

Our results are partly in line with a Swedish study (5), which found positive associations between fish intake of more than once per week during pregnancy and JIA risk, although our effect sizes were of substantially lower magnitude. The Swedish study did not specify portion sizes, complicating direct comparisons. Furthermore, our study specifically associates lean/semi-oily fish with increased JIA risk, while the Swedish study identified the strongest association with total fish intake without distinguishing between fish varieties (5).

We found no evidence of robust associations between exposure to dietary environmental contaminants and risk of JIA. This differs from the Swedish study which attributed the heightened risk of JIA to increased heavy metal exposure, including mercury, through fish intake (5), and another study showing that prenatal exposure to environmental contaminants can alter the cord serum metabolome, potentially increasing the risk of immune-mediated diseases such as JIA (42). Despite seafood accounting for 88% of total dietary mercury exposure – with lean fish contributing to more than half of this - as well as being a considerable source of other contaminants (20, 43, 44), we found no evidence that it contributed to JIA risk in MoBa. In fact, our sex-stratified analyses show an inverse relationship between exposure to POPs and JIA in girls. Unlike the Swedish study, which measured blood concentrations, our study relies on self-reported dietary data, but includes a much larger sample size (217 vs. 41 JIA cases) (5).

JIA is more prevalent in girls than boys (4), yet our study suggests that high seafood intake is more strongly associated with JIA risk in boys. Sex-stratified analyses showed no indication of increased risk of JIA when comparing high vs low intake of seafood and contaminant exposure (except lean/semi-oily fish and cadmium) in girls, on the contrary, estimates indicated a lower risk of JIA with high intake. In contrast, for boys, all associations indicated an increased risk of JIA.

Most studies on sex disparities in pediatric illnesses do not explore underlying causes (45), making our sex-stratified analyses valuable for addressing this knowledge gap. Although estrogen levels are often suggested as a cause for the higher prevalence of autoimmunity in women, the low and stable levels during childhood suggest other mechanisms (4). The varying patterns of JIA risk between boys and girls with seafood intake may be due to lack of statistical power given the sample size (girls, n = 139, boys, n = 78), and the results should be interpreted cautiously. The inverse relationship between POP exposure and JIA risk in girls observed in our study may not be directly linked to POPs, but could reflect a spurious association with oily fish, which was estimated to have a protective association in girls. This protective association may be related to nutrients in oily fish rather than POPs. A study on diabetes type 1 observed similar findings (46). A separate MoBa study on prenatal exposure to POPs showed immunosuppressive effects (32), which could potentially explain a protective association in girls. Inherent biological differences may also influence these sex-specific trends.

Gene-environment interaction analyses suggest that genetic predisposition modifies the effect of fish intake on JIA risk, and vice versa. Specifically, fish intake had a stronger estimated association with JIA risk in individuals with low genetic predisposition, while its impact was estimated as less pronounced in those with a high genetic risk. Our previous findings show that the PRS is more strongly associated with JIA in girls than in boys, with a higher proportion of female JIA cases having a standardized PRS >0 (submitted for publication)^1^. This might explain why we observe a stronger association between fish intake and JIA risk in boys, as male JIA cases, on average, have a lower genetic risk of JIA.

Our study’s strengths include its prospective design, comprehensive data collection with genetic liability, a large study population, and linkage to national registries, ensuring minimal loss to follow-up. A significant and novel strength is the incorporation of a PRS within a subset of our cohort, enabling us to study gene-environment interactions in JIA. By sex-stratification, we discerned variations in risk estimates between boys and girls. To our knowledge, this is the largest population-based prospective cohort study exploring environmental risk factors for JIA, identifying 217 cases.

While including more JIA cases than in previous studies, the sample size remains the main limitation of the study, as it reduces the power to detect small effects, especially in stratified analyses and for the subset with genotype data. We also lack data on JIA subtypes, which is important given the disease’s heterogeneity; different subtypes may have distinct pathomechanisms or vulnerabilities. We did not exclude controls with other systemic autoimmune diseases, potentially diluting the observed effects. Additionally, while the recruitment into MoBa was population-based, the cohort is not fully representative of the general population (47). For instance, the homogenous ethnic background of MoBa participants (48) may limit the generalizability of our findings to more diverse populations. The self-reported dietary data may result in exposure misclassification as the FFQ provide rough estimates, even though it has been validated (26). We cannot study exact dietary intake for the second half of the pregnancy as the FFQ was completed in week 22, however, we assume consistent dietary patterns throughout the pregnancy. Additionally, we lack measured blood concentration of contaminants. Our contamination estimates rely on broader Nordic averages rather than location-specific data, so this approach may not adequately capture exposure differences across Norway, especially in areas of higher contamination, highlighting the need for future research to measure blood concentrations. Although we adjusted for potential confounders, residual confounding cannot be ruled out due to the observational nature of the study. Lastly, since NPR data begins in 2008, JIA cases diagnosed and in remission between 2002-2008 may be missing. Some of the older-diagnosed JIA cases are also missing, because follow up ended in 2021.

In conclusion, we observed an increased risk of JIA in children whose mothers consumed high amounts of lean/semi-oily fish during pregnancy, particularly in boys. Despite lean fish being an important source of dietary mercury exposure, the heightened JIA risk was not explained by contaminant exposure in our study. Our findings also suggest a stronger association between fish intake and JIA in those with a low genetic predisposition to JIA. Further studies are warranted to explore the underlying mechanisms of seafood and JIA, as definitive causation cannot be inferred. This includes more precise assessments of contaminant exposure via blood samples, and the need to clarify the observed sex differences and genetic interactions.

## Data Availability

Data from the Norwegian Mother, Father and Child Cohort Study and the Medical Birth Registry of Norway used in this study are managed by the national health register holders in Norway (Norwegian Institute of public health) and can be made available to researchers, provided approval from the Regional Committees for Medical and Health Research Ethics (REC), compliance with the EU General Data Protection Regulation (GDPR) and approval from the data owners. The consent given by the participants does not open for storage of data on an individual level in repositories or journals. Researchers who want access to data sets for replication should apply through helsedata.no. Access to data sets requires approval from The Regional Committee for Medical and Health Research Ethics in Norway and an agreement with MoBa.

## Footnotes

^1^Haftorn KL, Rudsari HK, Jaholkowski PP, Dåstøl VØ, Hestetun SV, Andreassen OA, et al. *Nonlinearity and sex differences in the performance of a polygenic risk score for juvenile idiopathic arthritis*. (2024).
