# Supplementary material for: Maternal seafood intake, dietary contaminant exposure, and risk of juvenile idiopathic arthritis: exploring gene-environment interactions
Source: Front Immunol. 2025 Jan 14;15:1523990. doi: 10.3389/fimmu.2024.1523990 (PMC11772167; doi:10.3389/fimmu.2024.1523990)
Supplement: Supplementary file 1 [file Table1.docx]

|  | **All (controls n= 71,884, JIA cases n= 217)** | | **Boys (controls n= 36,784 and JIA cases n= 78)** | | **Girls (controls n= 35,100, JIA cases n= 139)** | |
| --- | --- | --- | --- | --- | --- | --- |
|  | **Unadjusted OR (95% CI)** | **aOR**^a^ **(95% CI)** | **Unadjusted OR (95% CI)** | **aOR**^a^ **(95% CI)** | **Unadjusted OR (95% CI)** | **aOR**^a^ **(95% CI)** |
| **High total fish intake** |  |  |  |  |  |  |
| <90^th^ percentile | Ref | Ref | Ref | Ref | Ref | Ref |
| ≥90^th^ percentile | 1.02  (0.65-1.58) | 1.00  (0.64-1.56) | 1.78  (0.98-3.23) | 1.78  (0.97-3.26) | 0.63  (0.32-1.24) | 0.62  (0.31-1.22) |
| **High lean/semioily fish intake** |  |  |  |  |  |  |
| <90^th^ percentile | Ref | Ref | Ref | Ref | Ref | Ref |
| ≥90^th^ percentile | **1.50**  **(1.03-2.20)** | 1.45 (0.99-2.18) | **2.13**  **(1.21-3.75)** | **2.02**  **(1.13-3.63)** | 1.18  (0.70-1.99) | 1.16  (0.68-1.99) |
| **High oily fish intake** |  |  |  |  |  |  |
| <90^th^ percentile | Ref | Ref | Ref | Ref | Ref | Ref |
| ≥90^th^ percentile | 0.81  (0.50-1.32) | 0.81  (0.50-1.33) | 1.45  (0.76-2.74) | 1.38  (0.72-2.65) | 0.49  (0.23-1.04) | 0.50  (0.23-1.08) |
| **High shellfish intake** |  |  |  |  |  |  |
| <90^th^ percentile | Ref | Ref | Ref | Ref | Ref | Ref |
| ≥90^th^ percentile | 1.12  (0.73-1.71) | 1.15  (0.75-1.75) | **1.83**  **(1.01-3.33)** | **1.87**  **(1.03-3.40)** | 0.76  (0.41-1.41) | 0.78  (0.42-1.45) |
| **High seafood intake** |  |  |  |  |  |  |
| <90^th^ percentile | Ref | Ref | Ref | Ref | Ref | Ref |
| ≥90^th^ percentile | 0.91  (0.58-1.49) | 0.90  (0.57-1.44) | 1.62  (0.87-3.00) | 1.62  (0.86-3.03) | 0.56  (0.27-1.13) | 0.55  (0.27-1.12) |

# Supplementary Table 1. Region of birth-adjusted associations between high vs. low seafood intake and JIA risk (overall and sex-stratified analyses).

^a^ Adjusted for maternal age, education, pre-pregnancy BMI, parity, daily caloric intake, history of inflammatory rheumatic disease in mother, parental smoking status during pregnancy, supplement use during pregnancy and region of birth. When lean/semioily fish is the main exposure, it is also adjusted for oily fish intake, and vice-versa.

# Supplementary Table 2 Region of birth-adjusted associations between high vs. low estimated dietary contaminant exposure and JIA risk (overall and sex-stratified analyses).

|  | **All (controls n= 71,884, JIA cases n= 217)** | | **Boys (controls n= 36,784 and JIA cases n= 78)** | | **Girls (controls n= 35,100, JIA cases n= 139)** | |
| --- | --- | --- | --- | --- | --- | --- |
|  | **Unadjusted OR (95% CI)** | **aOR**^a^ **(95% CI)** | **Unadjusted OR (95% CI)** | **aOR**^a^ **(95% CI)** | **Unadjusted OR (95% CI)** | **aOR**^a^ **(95% CI)** |
| **Mercury** |  |  |  |  |  |  |
| <90^th^ percentile | Ref | Ref | Ref | Ref | Ref | Ref |
| ≥90^th^ percentile | 0.91  (0.58-1.45) | 0.91  (0.57-1.46) | 1.17  (0.58-2.35) | 1.21  (0.59-2.46) | 0.77  (0.42-1.44) | 0.75  (0.41-1.42) |
| **Cadmium** |  |  |  |  |  |  |
| <90^th^ percentile | Ref | Ref | Ref | Ref | Ref | Ref |
| ≥90^th^ percentile | 1.39  (0.94-2.06) | 1.41  (0.93-2.13) | 1.47  (0.78-2.79) | 1.51  (0.76-2.97) | 1.35  (0.82-2.21) | 1.35  (0.80-2.28) |
| **Dioxins and dioxin-like (dl) compounds** |  |  |  |  |  |  |
| <90^th^ percentile | Ref | Ref | Ref | Ref | Ref | Ref |
| ≥90^th^ percentile | 0.86  (0.54-1.38) | 0.83  (0.51-1.35) | 1.46  (0.77-2.77) | 1.46  (0.75-2.85) | 0.56  (0.27-1.14) | 0.52  (0.25-1.08) |
| **Non-dioxin-like PCBs (PCB-153)** |  |  |  |  |  |  |
| <90^th^ percentile | Ref | Ref | Ref | Ref | Ref | Ref |
| ≥90^th^ percentile | 0.91  (0.58-1.45) | 0.90  (0.56-1.43) | 1.03  (0.49-2.14) | 1.02  (0.49-2.15) | 0.85  (0.47-1.54) | 0.83  (0.46-1.51) |

^a^ Adjusted for maternal age, education, pre-pregnancy BMI, parity, daily caloric intake, history of inflammatory rheumatic disease in mother, parental smoking status during pregnancy, supplement use during pregnancy and region of birth. When lean/semioily fish is the main exposure, it is also adjusted for oily fish intake, and vice-versa.

# Supplementary Table 3. Sex-stratified associations between fish consumption >300 grams per week and JIA risk.

| **Variables** | **Unadjusted OR (95% CI)** | **Adjusted OR* (95% CI)** |
| --- | --- | --- |
| **Both sexes****  <300 grams/week  ≥300 grams/week | Ref  1.15 (0.86-1.53) | Ref  1.16 (0.86-1.55) |
| **Boys*****  <300 grams/week  ≥300 grams/week | Ref  **1.91 (1.22-2.99)** | Ref  **1.92 (1.22-3.04)** |
| **Girls******  <300 grams/week  ≥300 grams/week | Ref  0.82 (0.56-1.22) | Ref  0.83 (0.56-1.23) |

JIA = juvenile idiopathic arthritis, OR = odds ratio, aOR = adjusted OR.

*aOR: adjusted for: maternal age, education, pre-pregnancy BMI, parity, daily caloric intake, history of inflammatory rheumatic disease in mother, parental smoking status during pregnancy and supplement use during pregnancy. When lean/semioily fish is the main exposure, it is also adjusted for oily fish intake, and vice-versa.

**For controls, n = 71,884. For JIA, n = 217
*** For controls, n =36,784. For JIA, n = 78
**** For controls, n = 35,100. For JIA, n = 139

# Supplementary Table 4. Overall and sex-stratified associations between seafood consumption and JIA, analyzed by quintiles.

|  | **All (controls n= 71,884, JIA cases n= 217)** | | **Boys (controls n= 36,784 and JIA cases n= 78)** | | **Girls (controls n= 35,100, JIA cases n= 139)** | |
| --- | --- | --- | --- | --- | --- | --- |
|  | **Unadjusted OR (95% CI)** | **aOR**^a^ **(95% CI)** | **Unadjusted OR (95% CI)** | **aOR**^a^ **(95% CI)** | **Unadjusted OR (95% CI)** | **aOR**^a^ **(95% CI)** |
| **High total fish intake** |  |  |  |  |  |  |
| 1 (0-17.1 g/day) | Ref | Ref | Ref | Ref | Ref | Ref |
| 2 (17.1-26.5 g/day) | 1.07  (0.71-1.62) | 1.10  (0.72-1.67) | 0.96  (0.45-2.05) | 0.96  (0.45-2.05) | 1.10  (0.67-1.81) | 1.14  (0.69-1.89) |
| 3 (26.5-35.9 g/day) | 0.93  (0.60-1.43) | 0.97  (0.63-1.50) | 1.01  (0.48-2.13) | 1.03  (0.49-2.18) | 0.88  (0.52-1.50) | 0.93  (0.55-1.59) |
| 4 (35.9-48.6 g/day) | 1.02  (0.67-1.56) | 1.06  (0.69-1.62) | 0.86  (0.40-1.86) | 0.87  (0.40-1.89) | 1.10  (0.66-1.82) | 1.15  (0.69-1.92) |
| 5 (48.6-351.7 g/day) | 1.02  (0.67-1.56) | 1.06  (0.69-1.62) | 1.80  (0.93-3.46) | 1.83  (0.94-3.57) | 0.65  (0.36-1.16) | 0.68  (0.38-1.22) |
| **High lean/semioily fish intake** |  |  |  |  |  |  |
| 1 (0-8.7 g/day) | Ref | Ref | Ref | Ref | Ref | Ref |
| 2 (8.7-14.7 g/day) | 0.98  (0.64-1.49) | 1.03  (0.67-1.57) | 1.02  (0.50-2.09) | 1.02  (0.50-2.10) | 0.94  (0.56-1.59) | 1.03  (0.61-1.74) |
| 3 (14.7-20.8 g/day) | 0.86  (0.56-1.33) | 0.92  (0.59-1.43) | 0.75  (0.34-1.63) | 0.74  (0.34-1.63) | 0.91  (0.54-1.54) | 1.02  (0.60-1.75) |
| 4 (20.8-28.7 g/day) | 0.95  (0.62-1.46) | 1.03  (0.67-1.59) | 1.21  (0.61-2.40) | 1.18  (0.59-2.38) | 0.82  (0.48-1.41) | 0.95  (0.55-1.66) |
| 5 (28.7-156.4 g/day) | 1.14  (0.76-1.71) | 1.20  (0.79-1.82) | 1.28  (0.65-2.52) | 1.23  (0.61-2.46) | 1.06  (0.64-1.76) | 1.21  (0.71-2.04) |
| **High oily fish intake** |  |  |  |  |  |  |
| 1 (0-1.9 g/day) | Ref | Ref | Ref | Ref | Ref | Ref |
| 2 (1.9-4.5 g/day) | 1.27  (0.84-1.91) | 1.29  (0.86-1.96) | 1.10  (0.49-2.50) | 1.09  (0.48-2.49) | 1.32  (0.82-2.12) | 1.36  (0.84-2.20) |
| 3 (4.5-7.3 g/day) | 1.22  (0.81-1.85) | 1.26  (0.83-1.92) | 1.64  (0.78-3.48) | 1.63  (0.76-3.50) | 1.06  (0.65-1.75) | 1.12  (0.67-1.87) |
| 4 (7.3-13.5 g/day) | 0.73  (0.46-1.17) | 0.75  (0.46-1.21) | 1.36  (0.63-2.97) | 1.32  (0.60-2.94) | **0.50  (0.27-0.93)** | **0.53  (0.28-1.00)** |
| 5 (13.5-249.4 g/day) | 1.07  (0.79-1.65) | 1.08  (0.69-1.68) | 2.00  (0.97-4.11) | 1.89  (0.89-4.01) | 0.74  (0.42-1.28) | 0.76  (0.43-1.35) |
| **High shellfish intake** |  |  |  |  |  |  |
| 1 (0-1.5 g/day) | Ref | Ref | Ref | Ref | Ref | Ref |
| 2 (1.5-3.7 g/day) | 1.24  (0.87-1.76) | 1.32  (0.92-1.88) | 1.52  (0.84-2.76) | 1.60  (0.87-2.91) | 1.11  (0.71-1.72) | 1.20  (0.77-1.87) |
| 3 (3.7-6.5 g/day) | 1.11  (0.77-1.60) | 1.18  (0.82-1.71) | 1.13  (0.59-2.17) | 1.17  (0.60-2.26) | 1.10  (0.71-1.71) | 1.19  (0.76-1.85) |
| 4 (6.5-266.3g g/day) | 1.01  (0.69-1.47) | 1.07  (0.73-1.57) | 1.61  (0.89-2.90) | 1.68  (0.93-3.04) | 0.74  (0.45-1.23) | 0.80  (0.48-1.32) |
| **High seafood intake** |  |  |  |  |  |  |
| 1 (0-19.8 g/day) | Ref | Ref | Ref | Ref | Ref | Ref |
| 2 (19.8-30.6 g/day) | 0.98  (0.64-1.48) | 1.01  (0.66-1.53) | 0.54  (0.24-1.22) | 0.54  (0.24-1.22) | 1.22  (0.74-2.00) | 1.27  (0.77-2.10) |
| 3 (30.6-41.4 g/day) | 0.80  (0.52-1.24) | 0.83  (0.54-1.30) | 0.53  (0.24-1.20) | 0.54  (0.24-1.22) | 0.96  (0.56-1.62) | 1.02  (0.60-1.73) |
| 4 (41.4-56.2g g/day) | 1.00  (0.66-1.51) | 1.04  (0.68-1.30) | 0.96  (0.48-1.89) | 0.97  (0.49-1.94) | 1.02  (0.61-1.71) | 1.07  (0.63-1.81) |
| 5 (56.2-668.0 g/day) | 1.04  (0.69-1.57) | 1.09  (0.72-1.65) | 1.60  (0.87-2.92) | 1.63  (0.87-3.03) | 0.71  (0.40-1.27) | 0.75  (0.42-1.35) |

^a^ Adjusted for maternal age, education, pre-pregnancy BMI, parity, daily caloric intake, history of inflammatory rheumatic disease in mother, parental smoking status during pregnancy and supplement use during pregnancy. When lean/semioily fish is the main exposure, it is also adjusted for oily fish intake, and vice-versa.

# Supplementary Table 5. Associations between seafood variables and JIA in dataset restricted to participants with available genetic data, n = 51,804.

|  | **All (controls n= 51,642, JIA cases n= 162)** | |
| --- | --- | --- |
|  | **Unadjusted OR (95% CI)** | **aOR**^a^ **(95% CI)** |
| **High total fish intake** |  |  |
| <90^th^ percentile | Ref | Ref |
| ≥90^th^ percentile | 1.15 (0.71-1.88) | 1.15 (0.70-1.89) |
| **High lean/semioily fish intake** |  |  |
| <90^th^ percentile | Ref | Ref |
| ≥90^th^ percentile | 1.44 (0.92-2.26) | 1.44 (0.91-2.27) |
| **High oily fish intake** |  |  |
| <90^th^ percentile | Ref | Ref |
| ≥90^th^ percentile | 0.80 (0.45-1.41) | 0.78 (0.44-1.39) |
| **High shellfish intake** |  |  |
| <90^th^ percentile | Ref | Ref |
| ≥90^th^ percentile | 1.50 (0.96-2.33) | 1.52 (0.97-2.36) |
| **High seafood intake** |  |  |
| <90^th^ percentile | Ref | Ref |
| ≥90^th^ percentile | 1.08 (0.65-1.78) | 1.08 (0.65-1.79) |

^a^Adjusted for: maternal age, education, pre-pregnancy BMI, parity, daily caloric intake, parental smoking status during pregnancy, and supplement use during pregnancy. When lean/semioily fish is the main exposure, it is also adjusted for oily fish intake, and vice-versa. **(controls n= 51,642, JIA cases n= 162)**

Supplementary Table 6. Interactions between seafood exposures and polygenic risk score (PRS)

| **Exposure** | **Unadjusted OR (95% CI, p-value)** | **Adjusted OR (95% CI, p-value)^a^** |
| --- | --- | --- |
| High total fish | **0.33**  **(0.12-0.89, 0.03)** | **0.33**  **(0.12-0.90, 0.03)** |
| High lean/semioily fish | 0.51  (0.20-1.30, 0.16) | 0.51  (0.20-1.30, 0.16) |
| High oily fish | 1.28  (0.33-4.88, 0.72) | 1.29  (0.34-4.92, 0.71) |
| High shellfish | 0.85  (0.32-2.22, 0.73) | 0.84  (0.32-2.20, 0.72) |
| High seafood | 0.48  (0.17-1.36, 0.17) | 0.49  0.17-1.38, 0.18) |

^a^Adjusted for: maternal age, education, pre-pregnancy BMI, parity, daily caloric intake, parental smoking status during pregnancy, supplement use during pregnancy, PRS and PCs 1-10. When lean/semioily fish is the main exposure, it is also adjusted for oily fish intake, and vice-versa. **(controls n= 51,642, JIA cases n= 162)**

# Supplementary Table 7. Associations between high seafood intake and high PRS in cases only.

| **Seafood variable (high vs low)** | **Unadjusted OR (95% CI)** | **aOR**^a^ **(95% CI)** |
| --- | --- | --- |
| Total fish | **0.33 (0.12-0.90)** | **0.17 (0.04-0.63)** |
| Lean/semioily fish | 0.53 (0.21-1.35) | 0.45 (0.16-1.32) |
| Oily fish | 1.31 (0.34-4.99) | 1.23 (0.21-7.15) |

^a^Adjusted for: maternal age, education, pre-pregnancy BMI, parity, daily caloric intake, parental smoking status during pregnancy, supplement use during pregnancy, PRS and PCs 1-10. When lean/semioily fish is the main exposure, it is also adjusted for oily fish intake, and vice-versa. **(JIA cases n= 162)**


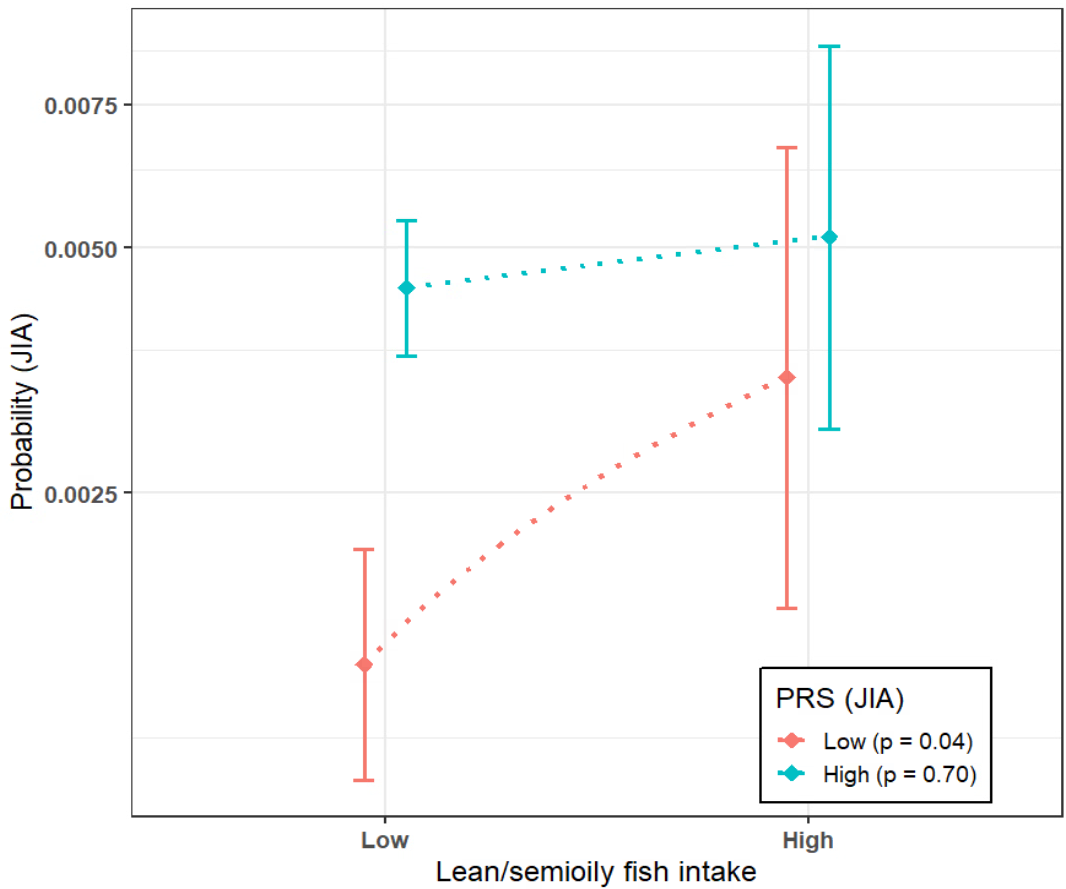


# Supplementary Figure 1. Association between lean/semioily fish intake and JIA risk grouped by high (=>0) and low (<0) polygenic risk score (PRS) for JIA. P-values indicate the significance of the associations between fish intake and JIA risk within each PRS group.


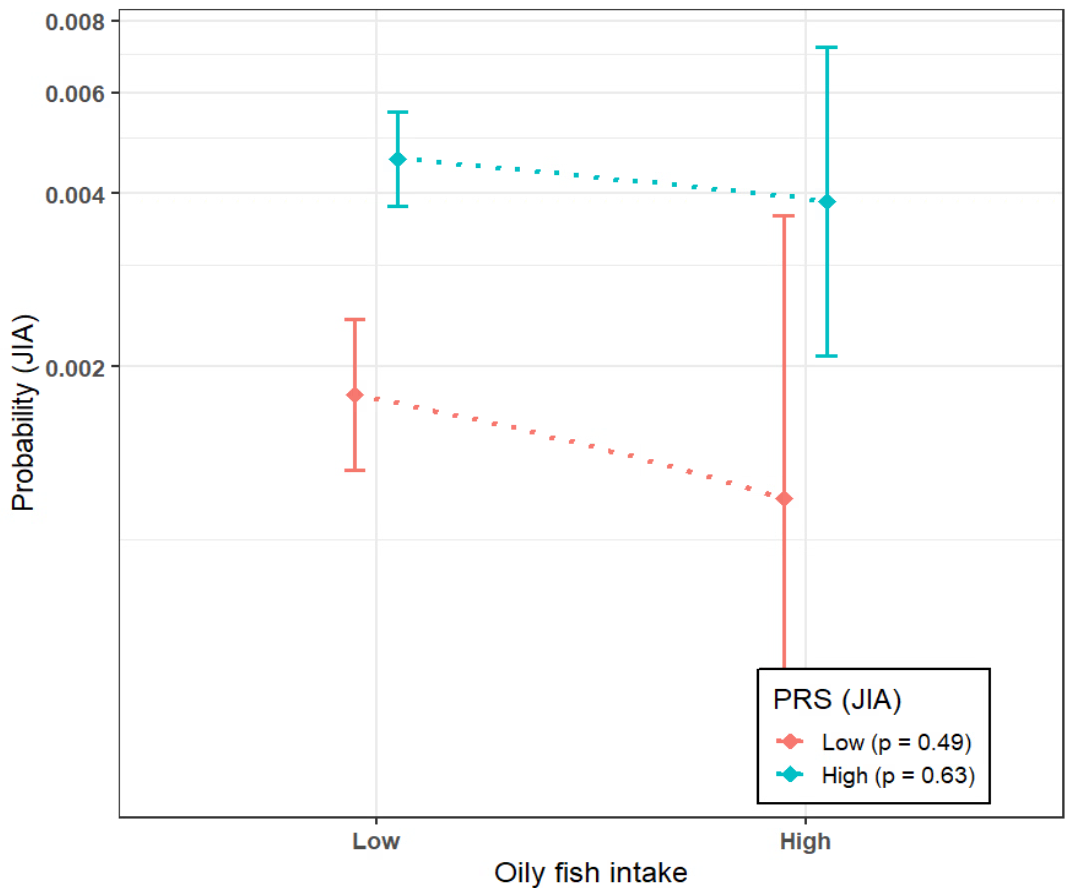


# Supplementary Figure 2: Association between oily fish intake and JIA risk grouped by high (=>0) and low (<0) polygenic risk score (PRS) for JIA. P-values indicate the significance of the associations between fish intake and JIA risk within each PRS group.

# Supplementary Table 8. Overall and sex-stratified associations between estimated dietary contaminant exposure and JIA, analyzed by quintiles.

|  | **All (controls n= 71,884, JIA cases n= 217)** | | **Boys (controls n= 36,784 and JIA cases n= 78)** | | **Girls (controls n= 35,100, JIA cases n= 139)** | |
| --- | --- | --- | --- | --- | --- | --- |
|  | **Unadjusted OR (95% CI)** | **aOR**^a^ **(95% CI)** | **Unadjusted OR (95% CI)** | **aOR**^a^ **(95% CI)** | **Unadjusted OR (95% CI)** | **aOR**^a^ **(95% CI)** |
| **Mercury** |  |  |  |  |  |  |
| 1 (0-0.012 ug/kg bw/day) | Ref | Ref | Ref | Ref | Ref | Ref |
| 2 (0.012-0.185 ug/kg bw/day) | 0.83  (0.53-1.27) | 0.85  (0.55-1.32) | 0.95  (0.45-2.01) | 0.99  (0.46-2.11) | 0.76  (0.45-1.29) | 0.79  (0.47-1.34) |
| 3 (0.185-0.253 ug/kg bw/day) | 1.11  (0.74-1.65) | 1.16  (0.77-1.74) | 1.08  (0.52-2.24) | 1.14  (0.54-2.39) | 1.12  (0.69-1.80) | 1.15  (0.71-1.88) |
| 4 (0.253-0.351 ug/kg bw/day) | 0.83  (0.54-1.27) | 0.87  (0.56-1.36) | 1.22  (0.60-2.48) | 1.34  (0.64-2.78) | 0.65  (0.38-1.13) | 0.67  (0.38-1.18) |
| 5 (0.351-0.363 ug/kg bw/day) | 0.95  (0.63-1.45) | 1.01  (0.65-1.56) | 1.36  (0.68-2.71) | 1.53  (0.74-3.17) | 0.78  (0.46-1.32) | 0.79  (0.46-1.37) |
| **Cadmium** |  |  |  |  |  |  |
| 1 (0-0.660 ug/kg bw/day) | Ref | Ref | Ref | Ref | Ref | Ref |
| 2 (0.660-0.822 ug/kg bw/day) | 1.02  (0.67-1.57) | 1.02  (0.66-1.59) | 0.86  (0.42-1.77) | 0.89  (0.42-1.90) | 1.13  (0.67-1.93) | 1.11  (0.64-1.93) |
| 3 (0.822-0.100 ug/kg bw/day) | 1.19  (0.79-1.80) | 1.21  (0.77-1.89) | 0.81  (0.39-1.68) | 0.87  (0.39-1.92) | 1.44  (0.87-2.38) | 1.40  (0.81-2.43) |
| 4 (0.100-0.130 ug/kg bw/day) | 0.83  (0.53-1.31) | 0.85  (0.51-1.41) | 1.01  (0.50-2.01) | 1.00  (0.50-2.44) | 0.73  (0.40-1.31) | 0.70  (0.36-1.37) |
| 5 (0.130-3.153 ug/kg bw/day) | 1.12  (0.74-1.70) | 1.14  (0.68-1.88) | 1.19  (0.61-2.31) | 1.31  (0.57-2.99) | 1.08  (0.63-1.84) | 1.03  (0.54-1.96) |
| **Dioxins and dioxin-like (dl) compounds intake** |  |  |  |  |  |  |
| 1 (0.060-0.369 pg/kg bw/day) | Ref | Ref | Ref | Ref | Ref | Ref |
| 2 (0.369-0.492 pg/kg bw/day) | 0.76  (0.50-1.16) | 0.76  (0.49-1.17) | 0.53  (0.24-1.20) | 0.57  (0.25-1.30) | 0.87  (0.53-1.43) | 0.84  (0.50-1.41) |
| 3 (0.492-0.628 pg/kg bw/day) | 0.88  (0.59-1.32) | 0.88  (0.57-1.35) | 0.94  (0.48-1.87) | 1.04  (0.50-2.15) | 0.85  (0.51-1.40) | 0.80  (0.47-1.37) |
| 4 (0.628-0.837 pg/kg bw/day) | 0.98  (0.66-1.45) | 0.97  (0.63-1.51) | 0.88  (0.44-1.77) | 1.00  (0.47-2.16) | 1.03  (0.64-1.66) | 0.94  (0.55-1.62) |
| 5 (0.837-22.40 pg/kg bw/day) | 0.72  (0.47-1.10) | 0.69  (0.42-1.12) | 1.22  (0.64-2.32) | 1.37  (0.65-2.91) | **0.46  (0.25-0.85)** | **0.40  (0.20-0.79)** |
| **Non-dioxin-like PCBs (PCB-153)** |  |  |  |  |  |  |
| 1 (0.030-0.439 pg/kg bw/day) | Ref | Ref | Ref | Ref | Ref | Ref |
| 2 (0.439-0.626 pg/kg bw/day) | 0.93  (0.62-1.42) | 0.95  (0.62-1.46) | 1.19  (0.53-2.66) | 1.31  (0.58-2.96) | 0.85  (0.52-1.38) | 0.84  (0.51-1.38) |
| 3 (0.626-0.873 pg/kg bw/day) | 1.00  (0.66-1.51) | 1.03  (0.67-1.58) | 1.55  (0.73-3.32) | 1.76  (0.80-3.87) | 0.82  (0.51-1.35) | 0.81  (0.49-1.37) |
| 4 (0.873-1.341 pg/kg bw/day) | 1.09  (0.73-1.62) | 1.13  (0.73-1.73) | 1.93  (0.93-4.00) | **2.24  (1.03-4.86)** | 0.82  (0.50-1.34) | 0.80  (0.47-1.35) |
| 5 (1.341-39.94 pg/kg bw/day) | 0.70  (0.44-1.09) | 0.71  (0.44-1.15) | 1.44  (0.67-3.09) | 1.67  (0.73-3.79) | **0.46  (0.26-0.84)** | **0.44  (0.23-0.83)** |

^a^ Adjusted for maternal age, education, pre-pregnancy BMI, parity, daily caloric intake, history of inflammatory rheumatic disease in mother, parental smoking status during pregnancy, supplement use during pregnancy and the child’s birth year
